# Antifungal and anti-virulence activities of cinnamon, thyme, and clove essential oils against *Candida* species

**DOI:** 10.3389/fphar.2026.1756267

**Published:** 2026-03-02

**Authors:** Islam Ahaik, Juan Carlos Nunez-Rodriguez, Sònia Abelló-Cros, Oscar Yanes, Samira Bouhdid, Toni Gabaldón

**Affiliations:** 1 Biotechnology and Applied Microbiology Team, Research Laboratory of Applied Chemistry and Microbiology and Biotechnology, Department of Biology, Faculty of Sciences, Abdelmalek Essaadi University, Tetouan, Morocco; 2 Institute for Research in Biomedicine (IRB Barcelona), The Barcelona Institute of Science and Technology, Barcelona, Spain; 3 Barcelona Supercomputing Centre (BSC-CNS), Barcelona, Spain; 4 Scientific and Technical Service, Rovira i Virgili University (URV), Tarragona, Spain; 5 Department of Electronic Engineering, Institut d'Investigació Sanitària Pere Virgili (IISPV), Rovira i Virgili University (URV), Tarragona, Spain; 6 Centro de Investigación Biomédica en Red de Diabetes y Enfermedades Metabólicas Asociadas (CIBERDEM), Instituto de Salud Carlos III (ISCIII), Madrid, Spain; 7 Catalan Institution for Research and Advanced Studies (ICREA), Barcelona, Spain; 8 CIBER de Enfermedades Infecciosas, Instituto de Salud Carlos III, Madrid, Spain

**Keywords:** antifungal resistance, *Candida*, essential oils, non-albicans *Candida*, virulence factors

## Abstract

**Background:**

*Candida* species are major opportunistic pathogens, with *Candida albicans* being the most frequent cause of candidiasis. However, increasing rates of non-*albicans* infections and antifungal resistance bring an urgent need for new therapeutics. Essential oils (EOs) have gained attention due to their potential to inhibit fungal growth and virulence.

**Methods:**

The chemical composition of cinnamon, thyme and clove EOs was analyzed by gas chromatography-mass spectrometry (GC-MS). Antifungal activity was evaluated against eighteen *Candida* strains representing nine species, including multidrug-resistant isolates. Minimum inhibitory concentrations (MICs) were determined. The two most active EOs were further assessed for their effects on germ tube formation and protease production, two key virulence traits.

**Results:**

GC-MS identified cinnamaldehyde, thymol and eugenol as the dominant components of cinnamon, thyme and clove EOs, respectively (> 70% relative abundance). All EOs displayed antifungal activity, with cinnamon and thyme being the most potent. Both oils showed increased activity against multidrug-resistant strains of *Candida parapsilosis* and *Nakaseomyces glabratus* compared with their susceptible parentals, suggesting they may target resistance trade-offs. All five clades of *Candidozyma auris* displayed low MICs for cinnamon EO (0.002-0.008% v/v), indicating high susceptibility. Cinnamon EO reduced germ tube formation in *C. albicans* from 97% to 12% at MIC/2, while thyme EO completely inhibited germ tube formation and induced pseudohyphae. Protease production was totally suppressed in *C. auris* clades II and III at MIC/2 thyme EO.

**Conclusion:**

These results highlight the strong dual activity of EOs, supporting further exploration of their potential as complementary therapeutic options against *Candida* infections.

## Introduction

*Candida* species are increasingly common fungal pathogens that pose a significant threat to immunocompromised individuals, particularly in the context of increasing resistance to currently available antifungals. In 2022, the World Health Organization (WHO) listed five *Candida* species among the critical and high-priority groups of fungal pathogens due to their high prevalence and rising drug resistance: *Candida albicans*, *Candidozyma auris* (*syn. Candida auris*), *Nakaseomyces glabratus* (syn. *Candida glabrata*), *Candida parapsilosis,* and *Candida tropicalis* (World Health Organization, 2022). With only four chemical classes of antifungals in clinical use—azoles, polyenes, echinocandins, and pyrimidine analogues—the growing incidence of drug-resistant *Candida* strains presents a therapeutic deadlock (Arastehfar et al., 2020). This situation severely limits treatment effectiveness, making candidiasis harder to manage and posing the threat of a silent pandemic. In light of this growing challenge, there is a pressing need to explore new therapeutic approaches, including the use of alternative antifungal agents (Kriegl et al., 2025), combination therapies (Soulaimani et al., 2021), and compounds targeting virulence rather than growth (Cui et al., 2015). By inhibiting virulence traits such as adhesion, production of hydrolytic enzymes, tissue invasion, and biofilm formation, candidate anti-virulence compounds could disarm the pathogen without necessarily killing it. This approach may reduce the selective pressure for resistance development, preserve the host’s microbiota, and minimize adverse side effects (Buroni and Chiarelli, 2020).

Recently, there has been a growing interest in discovering new and safe antimicrobial molecules derived from natural sources, particularly from aromatic and medicinal plants (Nazzaro et al., 2017). Essential oils (EOs) are complex mixtures of volatile plant-derived compounds that have demonstrated promising antifungal activity against a wide range of fungal pathogens, including *Candida* species (Kalemba and Kunicka, 2003; Tabassum and Vidyasagar, 2013; Nazzaro et al., 2017; Saracino et al., 2022). In this context, EOs derived from thyme, cinnamon and clove have attracted particular attention due to their strong activity against *Candida* species (Pina-Vaz et al., 2004; Pinto et al., 2009; Gucwa et al., 2018; Tran et al., 2020). Thyme EO is mainly characterized by the presence of thymol, cinnamon EO by cinnamaldehyde, and clove EO by eugenol (Yu, 2025). These phenolic compounds are known to exert potent antifungal effects, primarily through disruption of fungal cell membrane integrity, which may ultimately lead to fungal cell death (Ahmad et al., 2011; Shreaz et al., 2013; Niu et al., 2020; Didehdar et al., 2022).

Despite the broad antimicrobial potential of EOs, their inhibitory effects against clinically relevant antifungal-resistant strains, as well as their potential impact on *Candida* virulence factors, remain poorly understood.

Germ tube formation and the secretion of proteases are two major virulence factors in *Candida* species. Notably, the germ tube is a typical morphological feature of *C. albicans*, serving as a reliable marker for its identification. It consists of a small initial filamentous outgrowth from the yeast cell, indicating the start of hyphal formation. This morphological shift from yeast to hyphal form is crucial to the virulence of *C. albicans* (Mayer et al., 2013). Hyphal formation is associated with several pathogenic traits, including adherence to epithelial and endothelial surfaces, host tissue invasion, iron acquisition, escape from phagocytes, and immune evasion (Jacobsen et al., 2012). Another key virulence factor in *Candida* species is secreted proteases. These enzymes can break down a wide range of host proteins and are considered among the most critical contributors to pathogenicity. Proteases facilitate adhesion, promote tissue invasion, and contribute to host cell damage (Silva et al., 2014). Although the function of secreted proteases has been well characterized in *C. albicans*, their roles in non-*albicans* species remain underexplored.

In this study, we analyzed the composition of three commercial EOs—cinnamon, thyme and clove—and tested their inhibitory activity in comparison to established azole and echinocandin antifungals on a panel of eighteen *Candida* strains covering nine different species and including drug-resistant isolates. Furthermore, the impact of the two most effective EOs on key *Candida* virulence factors was investigated, focusing on germ tube formation in *Candida albicans* and protease production in non-*albicans Candida* species. Together, these approaches allowed us to assess not only the antifungal potential of essential oils but also their impact on key virulent traits, providing new insights into their possible role as alternative or complementary treatments against *Candida* infections.

## Materials and methods

### *Candida spp.* strains

A panel of eighteen fungal species covering different *Candida* species was used in this study, including wild type (WT), multidrug resistant (MDR), and clinical isolates (CL) (Table 1). WT strains refer to strains that have not undergone any laboratory-induced resistance selection and include both reference strains and clinical isolates that have not been exposed to resistance induction. MDR strains were previously generated in our laboratory from WT strains through controlled induction of resistance (REF). Clinical isolates correspond to strains recently recovered from patient specimens in Morocco (Ahaik et al., 2024). The panel included *C. albicans* (one wild type, one clinical isolate), *N. glabratus* (two reference strains, two multidrug resistant (MDR)), *C. parapsilosis* (one reference strain, one MDR), *C. tropicalis* (the reference strain), *Pichia kudriavzevii* (syn. *Candida krusei*, one clinical isolate), *C. dubliniensis* (one clinical isolate), *C. metapsilosis* (the reference strain), *C. orthopsilosis* (the reference strain), and *C. auris* (five reference strains). All the strains were initially streaked from glycerol stocks onto YPD plates and incubated for 48 h at 30 °C. For use in all subsequent assays, a single colony from each strain was then randomly chosen, preserved in 25% glycerol, and stored at −80 °C.

**TABLE 1:** List of *Candida* strains used in this study.

Strain code	Description	Resistance-linked mutations of MDR strains	More information
Calb_SC5314_WT	*Candida albicans* reference strain SC5314	​	(American Type Culture Collection, n.d)
Calb_CL	*Candida albicans* clinical isolate from vagina	​	Ahaik et al. (2024)
Cdub_CL	*Candida dubliniensis* clinical isolate from feces	​	Ahaik et al. (2024)
Ctro_CSPO_WT	*Candida tropicalis* reference strain CSPO	​	Mixão and Gabaldón, (2020)
Cmet_BP57_WT	*Candida metapsilosis* reference strain BP57	​	Pryszcz et al. (2015)
Cort_90-125_WT	*Candida orthopsilosis* reference strain 90-125	​	Riccombeni et al. (2012)
Cpar_CDC317_WT	*Candida parapsilosis* reference strain CDC317	​	Pryszcz et al. (2013)
Cpar_CDC317_MDR	*Candida parapsilosis* multidrug resistant, originated from Cpar_CDC317_WT, evolved in anidulafungin and switched to fluconazole	Missense in FKS1 and MRR1	(Nunez-Rodriguez et al., n.d)
Caur_I_WT	*Candida auris* clade I wild-type isolate from Pakistan (B8441)	​	Muñoz et al. (2018)
Caur_II_WT	*Candida auris* clade II wild-type isolate from Japan (B11220)	​	Muñoz et al. (2018)
Caur_III_WT	*Candida auris* clade III wild-type isolate from South Africa (MRU224)	​	Muñoz et al. (2018)
Caur_IV_WT	*Candida auris* clade IV wild-type isolate from Colombia	​	Muñoz et al. (2018)
Caur_V_WT	*Candida auris* clade V wild-type isolate from Iran (AR1097)	​	Muñoz et al. (2018)
Ngla_CBS138_WT	*Nakaseomyces glabratus* (syn. *Candida glabrata*) reference strain CBS 138	​	Carreté et al. (2018)
Ngla_CBS138_MDR	*Nakaseomyces glabratus* (syn. *Candida glabrata)* multidrug resistant, originated from Ngla_CBS138_WT, evolved in anidulafungin and switched to fluconazole	Frameshift/premature termination codon (PTC) in FKS1 Missense or inframe in FKS2 Stop loss in ERG3 PTC in IZH3 Missense in CSF1 and FKS3	Ksiezopolska et al. (2021)
Ngla_CST34_WT	*Nakaseomyces glabratus* (syn. *Candida glabrata*) reference strain CST34	​	Carreté et al. (2018)
Ngla_CST34_MDR	*Nakaseomyces glabratus* (syn. *Candida glabrata*) multidrug resistant, originated from Ngla_CST34_WT, evolved in anidulafungin and switched to fluconazole	Frameshift/PTC in FKS1 Missense or inframe in FKS2 Partial chromosome E duplication Missense in PDR1	Ksiezopolska et al. (2021)
Pkud_CL	*Pichia kudriavzevii* (syn. *Candida krusei*) clinical isolate from feces	​	Ahaik et al. (2024)

WT strains refer to strains without laboratory-induced resistance, including both reference strains and clinical isolates. MDR strains were generated from WT strains by directed experimental evolution. CL isolates were recovered from patient samples in Morocco. Strain codes, origins and known resistance-associated mutations of multidrug-resistant derivatives are listed.

### Essential oils

Three commercially available EOs were purchased from the Raihan cooperative (Zinat, Morocco): cinnamon (CEO), thyme (TEO), and clove (CLEO). According to the manufacturer, the aerial parts of thyme (both flowers and leaves), cinnamon bark, and clove buds were used as plant material to extract the essential oils of the three species by hydrodistillation. After extraction, the essential oils were separated from the aqueous distillate, and the oily layer was collected. The oils were then stored at 4 °C in amber glass vials, protected from light and air until use.

### Phytochemical composition using gas chromatography-mass spectrometry (GC-MS)

The chemical composition of the considered EOs was investigated by gas chromatography coupled to high-resolution mass spectrometry (GC-Exactive), following the procedure described by (Alshaikh and Perveen, 2021). Analyses were performed using a Thermo Scientific™ TRACE™ 1310 GC system coupled to an Exactive GC Orbitrap mass spectrometer, equipped with a Thermo Scientific™ TriPlus™ RSH fully automated sample preparation system. Compound separation was achieved on an HP-5MS capillary column (30 m × 0.25 mm × 0.25 µm; Agilent Technologies, Santa Clara, CA, USA). In brief, 10 µL of each EO was diluted in 990 µL of methanol (MeOH) to ensure complete solubilization and compatibility with the injection system. Diluted samples were then injected into a GC-Exactive system using helium as the carrier gas, with an inlet temperature of 250 °C, a split ratio of 20:1, and a column flow rate of 1.000 mL/min. A volume of 1 µL was injected. The GC’s initial temperature was maintained at 40 °C for 1.08 min, then increased at a rate of 3 °C/min to 240 °C, followed by a faster ramp of 30 °C/min up to a final temperature of 310 °C. The total run time was 70 min. Mass spectrometry was performed in full scan mode, and the acquisition range was 35–375 m/z. The chemical components in each essential oil were identified by comparing their mass spectra to the MS computer library (NIST), and retention indices were calculated using n-alkanes (C8-C20, from Sigma-Aldrich).

### Susceptibility of *Candida spp.* to essential oils

The susceptibility of *Candida* strains towards essential oils was evaluated using the Quantitative Phenotyping and Antimicrobial Susceptibility Testing method (Q-PHAST (Nunez-Rodriguez et al., 2025)). In brief, after growing the strain panel, four independent colonies were selected to represent four biological replicates per strain. Selected colonies were placed in a 96-well plate with 500 µL of YPD liquid and grown overnight at 35 °C in an orbital shaker (200 rpm), reaching culture saturation. The four replicates of each strain were distributed to minimize the possibility of cross-contamination. The saturated culture was diluted in sterile distilled water (3:200). Subsequently, a volume of 5 µL of diluted cells was spotted on previously prepared YPD agar plates containing a range of seven different concentrations of the three essential oils: 0.001%, 0.002%, 0.004%, 0.008%, 0.0156%, 0.0312%, and 0.0625% (v/v). Each essential oil was dissolved in DMSO and then mixed with YPD agar medium, maintaining a final DMSO concentration of 1% to minimize potential toxicity to fungal cells. Control plates containing the same amount of DMSO without EO were prepared in parallel. The susceptibility of the *Candida* panel to essential oils was compared to two antifungal drugs: fluconazole (FLZ) (Sigma-Aldrich, St. Louis, MO, USA) and anidulafungin (ANI) (Pfizer Pharmaceutical Group, New York, NY, USA) with a range of concentrations of 1, 2, 4, 8, 16, 32, and 64 μg/mL and 0.015, 0.03, 0.06, 0.25, 2, 4, and 8 μg/mL for FLZ and ANI, respectively. All plate transfers were carried out using the 96-channel PlateMaster (Gilson). Within a 35 °C incubator, the spotted agar plates were placed on scanners, where images were taken every 15 min throughout the 24-hour incubation period. Generated images together with metadata of strains and tested conditions were analyzed using the Q-PHAST graphical interface (https://github.com/Gabaldonlab/Q-PHAST). This software quantifies the growth of each spot and generates growth curves, from which the area under the curve (AUC) is calculated as an accurate fitness measure. AUC values obtained at each drug concentration were divided by the corresponding values measured under untreated conditions to calculate relative fitness (AUC_rel). MIC_50_ was defined as the concentration associated with a 50% reduction in relative growth to the untreated condition. Each MIC_50_ value represents the median of four biological replicates.

### Impact of EOs on germ tube production in *Candida albicans*

To assess the effect of EOs on germ tube formation, *C. albicans* SC5314 was cultured on YPD agar at 37 °C overnight to obtain fresh colonies. MIC and sub-inhibitory concentrations (½ MIC, ¼ MIC) of cinnamon and thyme EOs were prepared by first dissolving them in dimethyl sulfoxide (DMSO), followed by dilution in YPD broth to achieve the desired concentrations, ensuring the final DMSO content did not exceed 1% (v/v). For germ tube induction, fresh colonies were suspended in phosphate-buffered saline (PBS) and added to YPD broth supplemented with 10% horse serum and the corresponding EO concentration to obtain a final inoculum of 1 × 10^6^ cells/mL. Cultures were incubated at 37 °C with shaking for 3 h, including a growth control without EOs. Following incubation, a volume of 5 μL from treated and untreated suspensions was placed on sealed slides and examined by light microscope. The proportions of germ tubes, pseudohyphae, and yeast forms were determined from images by calculating 100 cells in each replicate. Data from the three biological replicates were reported as the percentage represented by each morphological form. Germ tubes were considered positive when they were observed to be emerging from yeast cells without any constriction at their starting point from the originating cell.

### Effect of EOs on secreted proteases activity in non-*albicans Candida* species

The protease activity in the *Candida* spp. panel was evaluated using the method described by (Dabiri et al., 2018) with some modifications. This method is based on the use of bovine serum albumin (BSA) as a protein substrate. 1 g of BSA is dissolved in 100 mL of sterile distilled water. The solution is sterilized by filtration and then added for a final concentration of 0.2% to a sterile culture medium containing dextrose (20 g/L), MgSO_4_ (0.5 g/L), KH_2_PO_4_ (1 g/L), and agar (20 g/L). The medium was then maintained at a temperature of 45 °C–50 °C. The corresponding EO concentrations were added to 50 mL Falcon tubes containing the BSA-supplemented medium. A single concentration, corresponding to half of the MIC (MIC/2) for each isolate, was used to evaluate the anti-protease effect of cinnamon and thyme essential oils. After homogenizing the mixture, the final solution is poured into sterile plates. A fungal inoculum was prepared using YPD liquid medium and incubated overnight at 35 °C. Approximately 5 × 10^6^ cells of each strain, suspended in 5 μL of sterile distilled water, were spotted onto BSA plates and incubated at 35 °C for 48 h. The activity of the proteases, estimated by calculating the Pz value, is defined as the ratio of the colony diameter to the sum of the colony diameter and the precipitation zone. Each strain was tested in triplicate. Lower Pz values correspond to higher protease production, while higher Pz values indicate lower enzymatic activity. The results were categorized into three levels: Pz = 1 indicated no enzymatic activity; Pz between 0.99 and 0.7 represented low to moderate activity, and Pz lower than 0.69 corresponded to high enzymatic activity.

### Statistical analysis

Unless indicated otherwise, all experiments were carried out in triplicate, with results presented as mean ± standard deviation (SD). Comparisons between the control and treatment groups were performed using Welch’s two-sample t-test in R (version 4.5.1). For the germ tube assay, the control group was compared with the MIC/2 and MIC/4 concentrations of cinnamon EO. For the protease production assay, the control group was compared with the MIC/2 concentrations of cinnamon and thyme EOs for each *Candida* strain. P-values were interpreted as follows: *p* < 0.05 (*), *p* < 0.01 (**), and *p* < 0.001 (***).

## Results

### Chemical composition of essential oils

We determined the chemical composition of cinnamon, thyme, and clove essential oils (EOs) using gas chromatography-mass spectrometry (GC-MS, see Materials and Methods). A total of 23 compounds were identified in thyme EO, making it the most chemically diverse among the three tested EOs, followed by clove EO, with 7 compounds, and cinnamon EO, with 5 compounds. Table 2 highlights the distinct chemical compositions of the three essential oils, reflecting differences in dominant compound classes and relative abundances of key constituents.

**TABLE 2:** Chemical profiles of cinnamon, thyme and clove EOs based on GC-MS analysis.

Essential oil	Compound name	tr (min)	% Area	Retention index	Chemical nature
Cinnamon	**Cinnamaldehyde**	25.21	**72.43**	1277	Phenylpropanoid
	Eugenol	28.84	10.37	1361	Phenylpropanoid
	Cinnamaldehyde dimethyl acetal	30.60	16.95	1400	Phenylpropanoid derivative
	2H-1-Benzopyran-2-one, 6-methyl-	36.92	0.24	1559	Coumarin derivative
Thyme	Alpha-pinene	9.4	0.76	929	Monoterpene
	3-carene	9.64	0.81	935	Monoterpene
	Beta-pinene	12.19	1.41	993	Monoterpene
	Terpinolene	13.26	1.64	1017	Monoterpene
	p-cymene	13.64	8.16	1027	Monoterpene
	Ciclohexane, 1-methylene-4-(1- methylethenyl)	13.80	0.44	1030	Monoterpene derivative
	ɣ-Terpinene	15.22	4.09	1061	Monoterpene
	5-Isopropyl-2-methylbicyclo[3.1.0]hexan2-ol	15.57	0.29	1069	Monoterpene alcohol
	alpha-Terpinene	16.56	0.18	1089	Monoterpene
	Linalool	17.18	1.6	1100	Monoterpene alcohol
	Borneol	20.14	0.15	1167	Monoterpene alcohol
	Terpinen-4-ol	20.69	0.53	1178	Monoterpene alcohol
	alpha-Terpineol	21.42	0.07	1193	Monoterpene alcohol
	trans-Verbenol	23.83	0.04	1247	Monoterpene alcohol
	Carvone	23.92	0.04	1250	Monoterpene ketone
	4-Terpinenyl acetate	25.69	0.04	1287	Monoterpene ester
	Carvacrol	25.79	0.05	1290	Monoterpene phenol
	**Thymol**	26.64	**74.6**	1308	Monoterpene phenol
	Phenol, 3-methyl-5-(1-methylethyl)-, methylcarbamate	29.45	0.17	1375	Aromatic carbamate
	Caryophyllene	31.33	4	1419	Sesquiterpene
	alpha-Humulene	32.71	0.16	1453	Sesquiterpene
	Caryophyllene oxide	37.82	0.57	1582	Sesquiterpene oxide
	Phenol, 3,5-diethyl-	49.98	0.21	1929	Alkylphenol
Clove	**Eugenol**	29.02	**81.74**	1365	Phenylpropanoid
	beta-Caryophyllene	31.36	14.46	1420	Sesquiterpene
	alpha-Humulene	32.72	1.71	1453	Sesquiterpene
	Isoeugenol acetate	35.78	1.07	1529	Phenylpropanoid ester
	Caryophylla-4(12),8(13)-dien-5α-ol	36.64	0.1	1552	Sesquiterpene alcohol
	Caryophyllenyl alcohol	37.31	0.14	1569	Sesquiterpene alcohol
	Caryophyllene oxide	37.82	0.78	1582	Sesquiterpene oxide

Tr = retention time (min); %Area = relative abundance. The main compound of each EO and its corresponding relative abundance are highlighted in bold.

Cinnamon EO was dominated by phenylpropanoids, which significantly contributed to the oil’s chemical profile. The main component of this EO was cinnamaldehyde, with a relative abundance of 72.43%, followed by cinnamaldehyde dimethyl acetal (16.95%) and eugenol (10.37%). Together, these three compounds represented the vast majority of the oil’s composition, highlighting the predominance of aldehyde and phenolic compounds in cinnamon EO. On the other hand, the main constituents of thyme EO were thymol (74.6%), *p*-cymene (8.16%), and ɣ-terpinene (4.09%). Other compounds that were identified in this EO belong to monoterpenes. Examples include alpha- and beta-pinene, *p*-cymene, ɣ-terpinene, and linalool. However, despite the presence of multiple monoterpene compounds, their overall contribution to the EO’s composition is relatively low. Instead, thymol, a monoterpene phenol, exhibits the highest relative abundance, dominating the oil’s composition. Clove EO was rich in eugenol (81.74%), beta-caryophyllene (14.46%), and alpha-humulene (1.71%). Eugenol, a phenylpropanoid, represented the major constituent and thus accounted for most of the chemical profile of the oil. In contrast, beta-caryophyllene and alpha-humulene, both belonging to the sesquiterpene class, were present in lower proportions but contributed to the overall composition of the oil.

### Susceptibility of *Candida* species to essential oils

**FIGURE 1:**
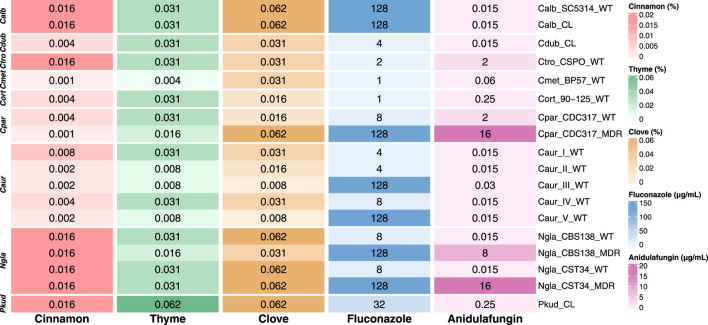
Comparative MIC_50_ profiles of *Candida* strains exposed to essential oils (% v/v) and antifungals (μg/mL). Strains are grouped by species: *C. albicans* (Calb), *C. dubliniensis* (Cdub), *C. tropicalis* (Ctro), *C. metapsilosis* (Cmet), *C. orthopsilosis* (Cort), *C. parapsilosis* (Cpar), *C. auris* (Caur), *N. glabratus* (Ngla), and *P. kudriavzevii* (Pkud). Color gradients represent the range of MIC values for each compound. Values of 128 and 16 were assigned to represent MIC >64 μg/mL and MIC >8 μg/mL for fluconazole and anidulafungin, respectively. Note that each color scale is adjusted to the specific range of each EO/drug (see text).

We used Q-PHAST to evaluate the antifungal activity of the tested agents, which allowed cost-effective, accurate susceptibility measurements while providing consistent results with the traditional broth microdilution assays (Nunez-Rodriguez et al., 2025). The minimum inhibitory concentrations MIC_50_, expressed as percentages (% v/v) for EOs and in micrograms per milliliter (μg/mL) for antifungal drugs, are summarized in Figure 1.

The MIC_50_ values for the three EOs ranged from 0.062% to 0.001% (v/v), with cinnamon EO demonstrating the most potent antifungal activity, characterized by the lowest range of MIC values (0.016%–0.001%); thyme EO showed intermediate values (0.031%–0.004%), and clove EO exhibited higher MICs (0.062%–0.008%) (Figure 1). Particularly, clove EO showed relatively higher MICs in a subset of strains, notably *C. albicans* (both Calb_SC5314_WT and Calb_CL), *N. glabratus* (Ngla_CBS138_WT, Ngla_CST34_WT, and its derivative Ngla_CST34_MDR), and *P. kudriavsevii* (Pkud_CL). Interestingly, certain multi-drug resistant strains showed increased susceptibility compared to their parental strains. For instance, *C. parapsilosis* Cpar_CDC317_MDR exhibited a four-fold MIC reduction to cinnamon EO relative to its parental strain (from 0.004% to 0.001%). A comparable pattern was observed for thyme and clove EOs, where the MICs decreased from 0.031% to 0.062% in Ngla_CBS138_WT to 0.016% and 0.031% in its resistant derivative, Ngla_CBS138_MDR. In contrast, the MIC for cinnamon EO remained unchanged, with both strains exhibiting 0.016%. Likewise, Ngla_CST34_MDR and its parental strain displayed identical MICs (0.016% for cinnamon, 0.031% for thyme, and 0.062% for clove). Both *C. parapsilosis* Cpar_CDC317_WT and *C. orthopsilosis* Cort_90-125_WT exhibited identical MIC values across all tested EOs. In contrast, *C. metapsilosis* Cmet_BP-57_WT was much more sensitive to cinnamon and thyme EOs, with very low MIC values of 0.001% and 0.004% (v/v), respectively. However, its response to clove EO was less considerable, with a MIC of 0.031% (v/v). Notably, despite the well-documented multidrug-resistant profile of *C. auris*, strains from this species displayed relatively low MIC values compared to other tested *Candida* species. Furthermore, EO susceptibility appeared to be clade-dependent. Clades I and IV exhibited higher MICs than other strains: 0.008% and 0.004% (v/v) for cinnamon EO and 0.031% for both thyme and clove, respectively.

Moreover, *C. albicans* (both the reference strain and the clinical isolate), along with its closely related species *C. dubliniensis* and *C. tropicalis*, exhibited similar MIC values of 0.031% (v/v) when exposed to thyme EO. In the case of cinnamon EO, *C. dubliniensis* appeared to be four times more sensitive (MIC of 0.004%) than the *C. albicans* and *C. tropicalis* strains (0.016%). In addition, both *C. albicans* strains showed higher MIC values (0.062%) than *C. dubliniensis* and *C. tropicalis* (0.031%) when tested against clove EO. *P. kudriavzevii* (Pkud_CL) showed strong resistance to both thyme and clove EOs, with MIC values corresponding to the highest tested concentration (0.062%). In contrast, it was more sensitive to cinnamon EO, with a MIC of 0.016%. Although clove EO displayed antifungal activity, its higher MIC values compared to cinnamon and thyme EOs indicated lower potency; therefore, it was not selected for subsequent virulence factor expression assays.

The MIC values of the two conventional antifungals varied considerably among tested *Candida* strains. Fluconazole MICs ranged from 1 to 64 μg/mL, while anidulafungin values ranged from 0.015 to 8 μg/mL. As expected, the multidrug-resistant strains displayed the highest MIC values: all three exceeded the tested fluconazole range (visualized as 128 μg/mL), while only Ngla_CST34_MDR and Cpar_CDC317_MDR exceeded the anidulafungin range (visualized as 16 μg/mL). Both clinical and reference strains of *C. albicans* showed elevated MIC values (128 μg/mL) when tested on fluconazole, highlighting the ability of *C. albicans* to grow at high fluconazole concentrations on solid media (Bordallo-Cardona et al., 2017).

### Essential oils’ effect on germ tube production in *C. albicans*

**FIGURE 2:**
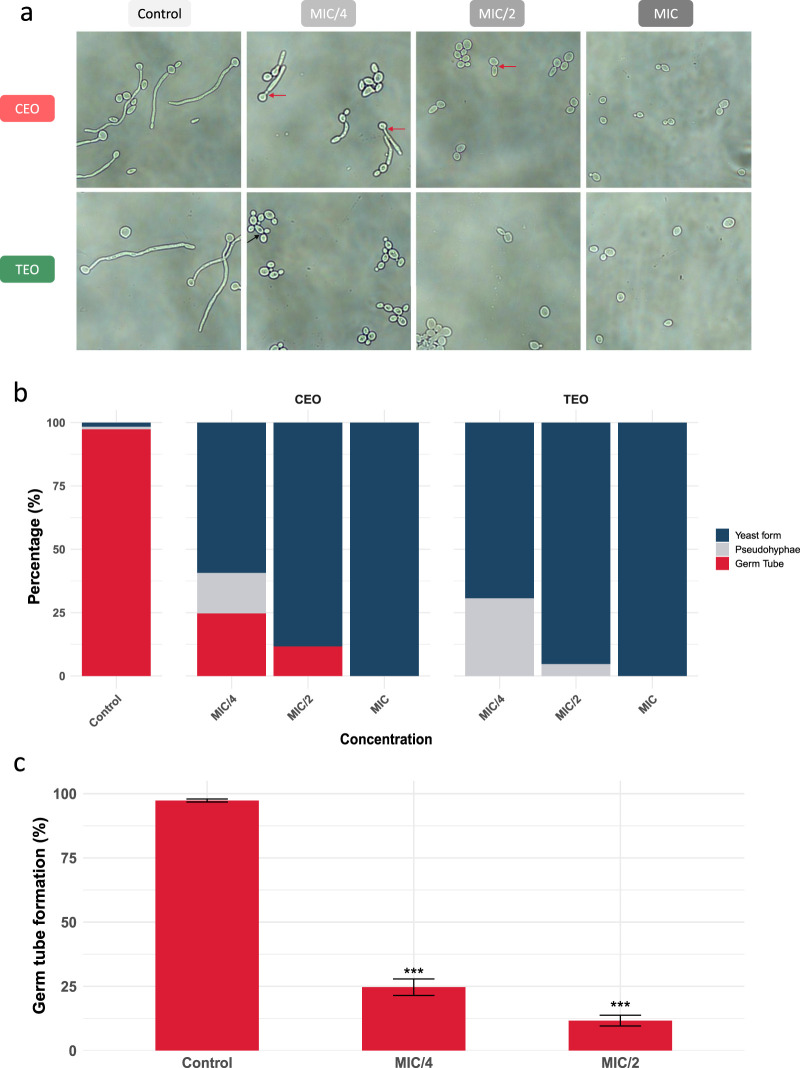
Effect of cinnamon and thyme EOs on germ tube formation in *C. albicans* SC5314. **(a)** Microscopic examination of *C. albicans* SC5314 germ tube under the effect of subinhibitory concentrations of cinnamon EO (CEO) and thyme EO (TEO) (magnification: ×40). The two essential oils exhibit different mechanisms of action on *C. albicans* germ tubes; cinnamon EO (CEO) tends more likely to shorten germ tube length, while thyme EO (TEO) completely inhibits hyphal production and promotes pseudohyphae formation. The red arrows indicate the absence of constrictions between the yeast and elongated cells, a characteristic feature of germ tube formation, whereas the black arrow shows the presence of constrictions, indicating pseudohyphal growth. **(b)** Morphological distribution of *C. albicans* SC5314 after exposure to cinnamon and thyme essential oils. Morphotypes (yeast form, pseudohyphae, and germ tubes) were quantified microscopically and expressed as a percentage of total cells. **(c)** Germ tube formation by *C. albicans* under the effect of sub-inhibitory concentrations (½ and ¼ MIC) of cinnamon EO, expressed as GTF/100 cells (mean ± SD, n = 3, ***p < 0.001).

We evaluated germ tube formation in *C. albicans* by counting 100 cells per sample with three independent replicates for each condition and categorizing each counted cell into yeast form, pseudohyphae, and germ tube-forming. We noted several differences between the two tested EOs. Most notably, cinnamon EO significantly reduced germ tube formation, decreasing from approximately 97% in the untreated control to 25% at MIC/4% and 12% at MIC/2 (Figures 2b,c). In addition, microscopic observation further revealed a notable shortening of germ tubes with increasing EO concentrations (Figure 2a). In contrast, thyme EO completely inhibited germ tube formation in all tested concentrations. This inhibition was coupled with an increase in the number of pseudohyphal cells, characterized by constrictions at the septal junctions and a more elongated yeast-like morphology, which indicates a morphological shift in response to thyme EO exposure. Overall, these observations suggest that cinnamon and thyme EOs interfere with *C. albicans* morphogenesis through distinct mechanisms, affecting both the amount and nature of filamentous growth.

### EOs’ effect on secreted protease activity

**FIGURE 3:**
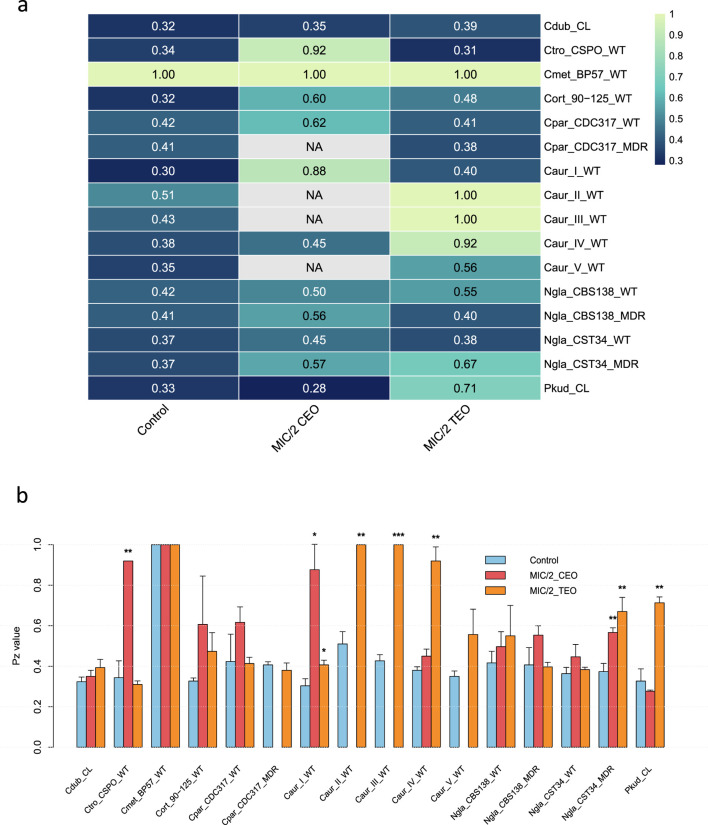
Protease production in *Candida* spp. under subinhibitory concentrations of cinnamon and thyme EOs. **(a)** Pz values of non-albicans *Candida* spp. and their variation under subinhibitory concentrations of CEO and TEO. Darker shades correspond to higher enzymatic activity (lower Pz values), while lighter shades indicate lower activity. “NA” values indicate conditions where protease activity data under CEO exposure were unavailable for Cpar_CDC317_MDR, Caur_II_WT, Caur_III_WT and Caur_V_WT due to their very low MIC/2 values. **(b)** Statistical comparison of protease activity under CEO and TEO exposure in *Candida* species. Bar plots represent the mean Pz values ± from three biological replicates. Blue bars correspond to the untreated controls, red bars to MIC/2 cinnamon essential oil (CEO), and orange bars to MIC/2 thyme essential oil (TEO). Statistical comparisons were performed between control and treatment groups using Welch’s t-test. Significance is indicated as follows: *p* < 0.05 (*), *p* < 0.01 (**), *p* < 0.001 (***).

The variation in protease activity among *Candida* species under control and subinhibitory treatment conditions is presented in Figure 3a. To confirm observed differences between treatments and between species, we performed Welch’s *t*-test for pairwise comparisons between control and MIC/2 treatments of CEO and TEO, identifying strains with significant changes in protease activity (Figure 3b).

Most of the tested strains were identified as strong producers of secreted proteases under growth control conditions, with Pz values ranging between 0.30 and 0.51. Only *C. metapsilosis* (Cmet_BP-57_WT) was unable to produce the secreted proteases, exhibiting a Pz value of 1. Exposure to MIC/2 concentrations of cinnamon EO led to noticeable decreases in secreted protease activity in several *Candida* strains. For instance, *C. tropicalis* (Ctro_CSPO_WT) and *C. auris* clade I (Caur_I_WT) showed significant drops in activity, from 0.34 to 0.92 (*p* < 0.01) and from 0.30 to 0.88 (*p* < 0.05), respectively. In *N. glabratus*, the two MDR strains exhibited a moderate reduction in protease activity compared to their corresponding wild-type strains. For example, Ngla_CBS138_MDR increased from 0.41 to 0.56, compared to a smaller change in Ngla_CBS138_WT (0.42–0.5). Similarly, Ngla_CST34_MDR showed a greater increase in Pz value (0.37–0.57) than Ngla_CST34_WT (0.37–0.45). These results suggest that protease production in multidrug-resistant isolates of *N. glabratus* is more sensitive to cinnamon EO treatment than in their wild-type parentals. Interestingly, *P. kudriavzevii* (Pkud_CL) was the only strain that exhibited enhanced protease production in the presence of cinnamon EO at sub-inhibitory concentration, as indicated by a decrease in its Pz value from 0.33 (growth control) to 0.27. This suggests a potential stress-induced upregulation of secreted protease activity in response to this EO. The remaining strains (Cort_90-125_WT, Cpar_CDC317_WT, and Caur_IV_WT) showed only slight increases in their Pz values when compared to their corresponding growth controls. Protease activity data under cinnamon EO exposure were not available for the strains Cpar_CDC317_MDR, Caur_II_WT, Caur_III_WT and Caur_V_WT, as they exhibited very low MIC/2 values.

On the other hand, the response to thyme EO exposure in terms of protease activity varied among *Candida* species. A particularly striking effect was observed in *C. auris* clades II and III (Caur_II_WT and Caur_III_WT), where protease activity was completely suppressed (Pz = 1.00; *p* < 0.01 and *p* < 0.001, respectively). Additionally, a strong reduction in protease activity was observed in Caur_IV_WT, with a Pz value of 0.92 (*p* < 0.01). In contrast, clade I and V showed only slight increases in Pz values compared to their untreated control, indicating low to mild inhibition. Among *N. glabratus* strains, Ngla_CST34_MDR showed significant inhibition of protease activity, with Pz values increasing from 0.37 (growth control) to 0.67 upon treatment (*p* < 0.01), compared to its wild-type parental strain (Ngla_CST34_WT), whose Pz remained nearly unchanged. This observation points to an enhanced vulnerability of the resistant strain to thyme EO, indicating a potential cross-sensitivity between standard drugs and EOs (Gabaldón, 2023). Notably, *C. tropicalis* and *P. kudriavzevii* exhibited opposite responses in the two EOs. For instance, Pkud_CL displayed a marked increase in its Pz value when exposed to thyme EO (from 0.33 to 0.71; *p* < 0.01), reflecting strong inhibition of enzyme activity. In contrast, Ctro_CSPO_WT showed a slight decrease in Pz value, from 0.34 (control) to 0.31 under thyme EO treatment, suggesting a modest enhancement in protease production. These findings emphasize that both cinnamon and thyme EOs have the ability to modulate protease activity in *Candida* species in a species- and strain-dependent manner, suggesting their potential as species-specific anti-virulent agents.

## Discussion

The fight against fungal infections is becoming more challenging as the armamentarium of available antifungals remains limited and resistance continues to increase. This scenario poses a pressing need to develop new antifungal compounds or alternative therapeutic approaches. In recent years, EOs have attracted interest due to their long history of use for their antimicrobial properties in treating viral, bacterial, and fungal infections (Reichling et al., 2009). Additionally, they have been investigated for use in topical creams (Nejati et al., 2015), as food preservatives (Tongnuanchan and Benjakul, 2014), and as biocides in sustainable agriculture (Assadpour et al., 2024). The present study focused on investigating the antifungal and anti-virulence potential of three EOs against a panel of eighteen diverse *Candida* strains, including reference, clinical, and multidrug-resistant strains from nine different species.

GC-MS analysis revealed that the major components of cinnamon, thyme, and clove EOs were cinnamaldehyde (72.43%), thymol (74.6%), and eugenol (81.74%), respectively. They belong to two major classes of bioactive molecules—phenylpropanoids (cinnamaldehyde and eugenol) and monoterpenes (thymol). Their high relative abundance indicates that these compounds likely play a central role in the biological activity of each oil. Previous GC-MS studies have shown that cinnamon and clove EOs may consist of a limited number of components. For instance, cinnamon EO was reported to contain only five identified constituents, with cinnamaldehyde as the major compound (96.80%) (Keshvari et al., 2013), while clove EO exhibited a similarly simple profile, with four main compounds dominated by eugenol (74.34%) (Trifan et al., 2021). In the case of cinnamon EO, the predominance of cinnamaldehyde aligns with previous findings in *Cinnamomum zeylanicum*, as reported by (Saleem et al., 2015; Shahina et al., 2018; Tran et al., 2020), where cinnamaldehyde was identified as the major compound. In the case of thyme EO, the high content of thymol is consistent with earlier studies in *Thymus vulgaris* (Hudaib et al., 2002; Borugă et al., 2014; Baj et al., 2020). Similarly, the clove EO analyzed in this study showed a dominant presence of eugenol, which is consistent with the characteristic chemical profile of *Syzygium aromaticum* described in previous works (Didehdar et al., 2022; Kiki, 2023; Momo et al., 2024). Overall, these findings confirm the consistency of our results with previously reported chemical profiles of the considered EOs while also highlighting slight differences that can be associated with factors such as geographic origin, plant chemotype, extraction conditions, and seasonal variation.

In this study, MIC values for cinnamon, thyme and clove EOs were expressed in % (v/v), as this unit remains the most widely used in the literature. The results revealed distinct inhibition responses to EOs, with cinnamon EO showing the strongest effect, followed by thyme and clove EOs. The strong antifungal activity of cinnamon EO is mainly attributed to cinnamaldehyde, which acts by disturbing the ergosterol biosynthesis pathway and directly binding to ergosterol within the fungal membrane, thus weakening membrane integrity (da Nóbrega Alves et al., 2020; Khan et al., 2013). In addition, cinnamaldehyde and related compounds strongly inhibit plasma membrane ATPase activity, which interferes with ATP-dependent efflux processes (Shreaz et al., 2013; Shreaz et al., 2011). In addition, cinnamon EO can also induce spindle defects and G2/M cell cycle arrest in *C. albicans*, highlighting its potential to disrupt fungal growth through multiple cellular targets (Shahina et al., 2018). Similarly, thyme EO, rich in thymol, exhibited moderate to strong inhibitory effects, in agreement with earlier reports highlighting its ability to alter ergosterol and lipid organization, increasing permeability and causing cytoplasmic leakage (Ahmad et al., 2011; de Castro et al., 2015). While both compounds share membrane-disrupting activities, thymol additionally induces mitochondrial dysfunction, reactive oxygen species accumulation, and vacuolar/cytoplasmic disorganization, highlighting distinct effects on fungal cell structure (Braga and Ricci, 2011). Clove EO was generally less potent than cinnamon and thyme EOs; however, it still showed antifungal activity mainly attributed to eugenol, which disrupts the fungal membrane, causes ion and macromolecule leakage, and induces oxidative stress that weakens mitochondrial function and cellular homeostasis in *Candida* species (Didehdar et al., 2022).

Among the tested MDR strains, *C. parapsilosis* Cpar_CDC317_MDR showed the most pronounced sensitivity to EOs, with a fourfold decrease in MIC for CEO and a twofold decrease for TEO compared to its parental strain (Cpar_CDC317_WT). These reductions occurred despite the presence of mutations in FKS1 and MRR1, which confer echinocandin resistance by reducing β-1,3-glucan synthase binding and azole resistance via expression of efflux pumps, respectively (Nunez-Rodriguez et al. n.d.). Similarly, *N. glabratus* Ngla_CBS138_MDR exhibited a twofold decrease in MIC values for thyme and clove EOs relative to its parental strain (Ngla_CBS138_WT), even though it carries mutations in FKS1-3, ERG3, IZH3 and CSF1. These mutations contribute to echinocandin resistance through altered glucan synthase activity, and azole resistance via ergosterol biosynthesis changes, and affect cell wall integrity and zinc homeostasis (Ksiezopolska et al., 2021). In contrast, *N. glabratus* Ngla_CST34_MDR, which harbors mutations in FKS1, FKS2 and PDR1, along with a partial duplication in chromosome E, displayed MIC values similar to its parental strain (Ngla_CST34_WT) across all tested EOs. These alterations confer echinocandin resistance via changes in β-1,3-glucan synthase and azole resistance through increased efflux pump activity (Ksiezopolska et al., 2021). Overall, these findings suggest that EOs act through mechanisms distinct from those of conventional antifungal drugs and may overcome classical resistance mechanisms, highlighting the need for further investigation into their specific molecular targets and pathways.

Interestingly, *C. auris*, known for its global spread and emerging resistance, showed notable susceptibility to the tested EOs, with comparatively lower MICs than other *Candida* species. In particular, clades II, III and V were the most sensitive. In contrast, clades I and IV exhibited higher MICs, indicating reduced susceptibility. A recent comparative review reported considerable variation in antifungal resistance; clades I, III, and IV are characterized by multidrug resistance, particularly to fluconazole and amphotericin B, which complicates treatment, whereas clades II, V and VI show much lower resistance (da Silva et al., 2025). Moreover, the expression of virulence factors such as adhesion expression, biofilm formation and hydrolytic enzyme production are generally higher in the more outbreak-prone clades (I, III, and IV) compared to the less resistant clades (Kim et al., 2024; Santana et al., 2024).

*C. albicans* (both Calb_SC5314_WT and Calb_CL), *C. tropicalis* (Ctro_CSPO_WT), and *C. dubliniensis* (Cdub_CL), three closely related species, exhibited similar MICs for TEO, with *C. dubliniensis* being more susceptible to CEO. Comparative studies have shown that the three species often display similar susceptibilities to EOs, despite the presence of species-specific differences (Pozzatti et al., 2008; Pozzatti et al., 2010; Mandras et al., 2016). *C. parapsilosis* (Cpar_CDC317_WT) and its sibling species *C. orthopsilosis* (Cort_90-125_WT) followed the same trend, reinforcing the idea that phylogenetically related species tend to respond similarly to EOs. However, *C. metapsilosis* (Cmet_BP57_WT) displayed lower MIC values when exposed to CEO and TEO, indicating greater sensitivity and suggesting that even small genetic differences within the complex can lead to distinct responses. *P. kudriavzevii* (Pkud_CL) exhibited the highest MICs for both CEO and TEO. Although classified in the WHO fungal priority pathogens list as a medium-priority species, it demands further investigation for new antifungals. *P. kudriavzevii* is intrinsically resistant to fluconazole, and recent reports have documented emerging resistance to other antifungal agents as well (Badiee et al., 2017; Jamiu et al., 2021; Zhang et al., 2023). Taken together, these results reinforce the idea that CEO and TEO, rich in cinnamaldehyde and thymol, possess notable antifungal activity, particularly against strains less responsive to azoles. Their role as complementary agents in managing resistant *Candida* infections merits further exploration.

Both cinnamon and thyme EOs disrupted *C. albicans* filamentation but through different effects. Cinnamon EO mainly shortened and partially inhibited germ tubes, while thyme EO fully hindered true hyphae, a key virulence factor in *C. albicans*, and promoted pseudohyphae formation. These differences likely result from distinct mechanisms; however, the molecular basis of these contrasting effects is still unknown.

Cinnamaldehyde has been reported to decrease germ tube formation, along with adhesion and hydrolytic enzyme production, collectively weakening virulence factors of *C. albicans* (Pootong et al., 2017; El-Baz et al., 2021; Essid et al., 2019). Previous studies reported that cinnamon EO inhibited germ tube formation in *C. albicans* isolates, with inhibition percentages ranging from 44.7% to 82.9% at concentrations of 62.5-500 μg/mL (Carvalho et al., 2018). At subinhibitory concentrations (62.5 and 31.25 μg/mL), cinnamaldehyde significantly reduced germ tube formation in a dose-dependent manner (Pootong et al., 2017). Additionally, both cinnamon bark and clove EOs, containing cinnamaldehyde and eugenol, inhibited hyphal morphogenesis, leading to substantial reductions in germ tube formation (Shahina et al., 2022). In contrast, thyme EO has been shown to promote pseudohyphal structures in *C. albicans*; a study observing morphological changes reported that exposure to thyme (and tea tree) oils led to noticeable alterations in colony and cell morphology, particularly including pseudohyphae appearance (Rajkowska et al., 2014). Furthermore, Alshaikh et al. reported that thyme EO inhibited germ tube formation by over 80% in fluconazole-resistant strains at ½ MIC (Alshaikh and Perveen, 2021). Thymol was found to significantly reduce hyphal formation in a dose-dependent manner at sub-inhibitory concentrations: at 31.25 μg/mL, around 46% of cells formed germ tubes, while at 62.5 μg/mL, only 29% were able to produce them (Braga et al., 2007).

Another virulence factor in *Candida* species is the protease production, an important group of hydrolytic enzymes contributing to their pathogenicity. *C. albicans* possesses a well-characterized family of ten genes (secreted aspartyl proteases; SAP1-SAP10) with roles linked to yeast growth, hyphal invasion, and stress adaptation (Naglik et al., 2003). Non-*albicans Candida* species also produce secreted proteases, but with fewer members or different types (Tóth et al., 2019; Zaugg et al., 2001; D Gilfillan et al., 1998). For instance, *C. dubliniensis, C. tropicalis*, and *C. parapsilosis* encode SAP homologs with varying sequence similarity and expression profiles, while *N. glabratus* lacks classical SAPs and instead produces yapsins (YPS1-YPS11), which are glycosylphosphatidylinositol (GPI)-anchored aspartyl proteases involved in cell wall remodeling and host interaction (Kaur et al., 2007; Pandey et al., 2018). In contrast, emerging species such as *C. auris* carry SAP gene homologues (Jenull et al., 2021; Kim et al., 2023; Chatterjee et al., 2015), though their exact functions in virulence remain under investigation. Interestingly, protease production in *C. auris* differs between clades, which was previously demonstrated between clades I and III, with the former showing greater virulence in infection models (Wang et al., 2018; Fan et al., 2021). To our knowledge, this is the first study comparing the five clades of *C. auris* in terms of protease activity and evaluating the effect of EOs on it.

Cinnamon and thyme EOs’ effects on protease activity revealed distinct patterns across the tested *Candida* species, with several strains showing marked reductions in protease production. For instance, Caur_I_WT and Ctro_CSPO_WT showed a strong decrease in their protease activity when exposed to cinnamon EO, while Caur_II_WT and Caur_III_WT displayed total inhibition when treated with thyme EO. In addition, Caur_IV_WT and Pkud_CL exhibited a significant reduction in their protease activity. Another noteworthy finding was observed in Ngla_CST34_MDR, where protease activity was notably affected by both oils compared to its parental strain. Even though high antifungal MICs were needed, inhibiting virulence factors in such strains is a valuable strategy because it can diminish their pathogenic potential. *C. metapsilosis* has often been reported as unable to produce detectable levels of proteases, a feature that may be influenced by strain differences or methodological factors (Neji et al., 2017; Gago et al., 2014; Sabino et al., 2011). Compared with *C. parapsilosis sensu stricto*, relatively few studies have focused on the enzymatic activities of *C. orthospilosis* and *C. metapsilosis*, leaving important gaps in our understanding of their virulence potential. Nevertheless, these species should not be underestimated, as they are increasingly identified as emerging pathogens capable of acquiring antifungal resistance and causing severe clinical infections (Del Olmo et al., 2025; Del Olmo and Gabaldón, 2024). In a few cases, such as *C. tropicalis* exposed to thyme EO or *P. kudriavzevii* exposed to cinnamon EO, we observed a slight increase in secreted protease activity at sub-inhibitory concentrations, which was demonstrated by the decrease of the Pz value in comparison to the control. This may indicate an adaptive stress that helps to control virulence in the presence of bioactive molecules. Our findings show evidence that exposure to subinhibitory concentrations of EOs can reduce and even suppress protease expression in *Candida* species. Most previous studies have focused on *C. albicans* (Pootong et al., 2017; Khan et al., 2014; Shreaz et al., 2012), while data on non-*albicans Candida* species remain limited. To our knowledge, this study provides the first comparative assessment of protease expression in response to EOs across multiple non-*albicans Candida* species.

## Conclusion

Our study provides compelling evidence of potent antifungal activity of EOs against a broad range of *Candida* species and strains, extending earlier results and underscoring their potential as alternative or complementary therapeutic options. Furthermore, our results suggest that EOs can weaken pathogenicity even without the complete elimination of fungal cells, as evidenced by their ability to inhibit germ tube formation in *C. albicans* and reduce secreted protease activity in non-*albicans* species. Importantly, the strong activity of EOs across diverse isolates indicates a prospect not only for treating a broad range of fungal infections but also a potential to help control multidrug-resistant strains in healthcare settings. Nevertheless, further investigations are necessary to translate these promising *in vitro* results into clinical applications, including toxicity assessment in suitable animal models such as *Galleria mellonella* and the optimization of delivery systems through advanced formulations like nanoparticles to increase stability, bioavailability, and therapeutic efficacy.

## Data Availability

The original contributions presented in the study are included in the article/supplementary material, further inquiries can be directed to the corresponding author.
